# Supplementary material for: Abrupt weakening of deep Atlantic circulation at the last glacial inception
Source: Nat Commun. 2025 Aug 14;16:7555. doi: 10.1038/s41467-025-62960-y (PMC12354891; doi:10.1038/s41467-025-62960-y)
Supplement: Supplementary file 1 — Supplementary Information [file 41467_2025_62960_MOESM1_ESM.pdf]

Abrupt weakening of deep Atlantic circulation at the last glacial inception

Supplementary Information

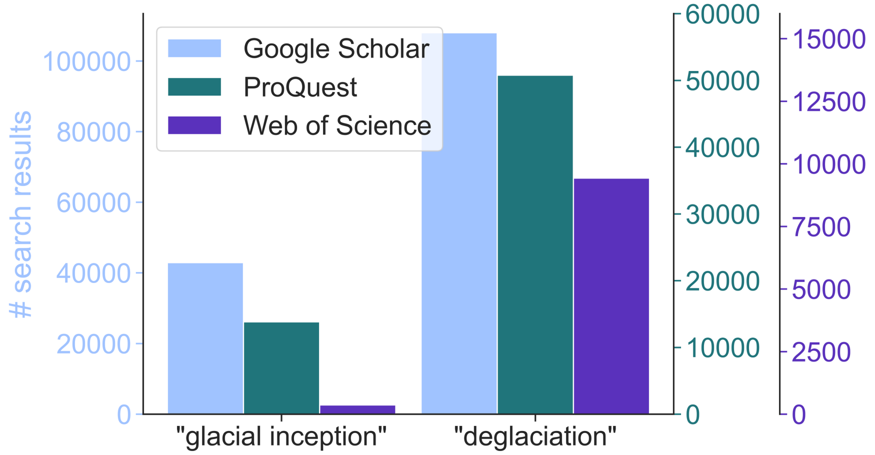

Supplementary Fig. 1. Numbers of search results returned using Google Scholar™, ProQuest™, and Web of Science™ for the terms "glacial inception" and "deglaciation".

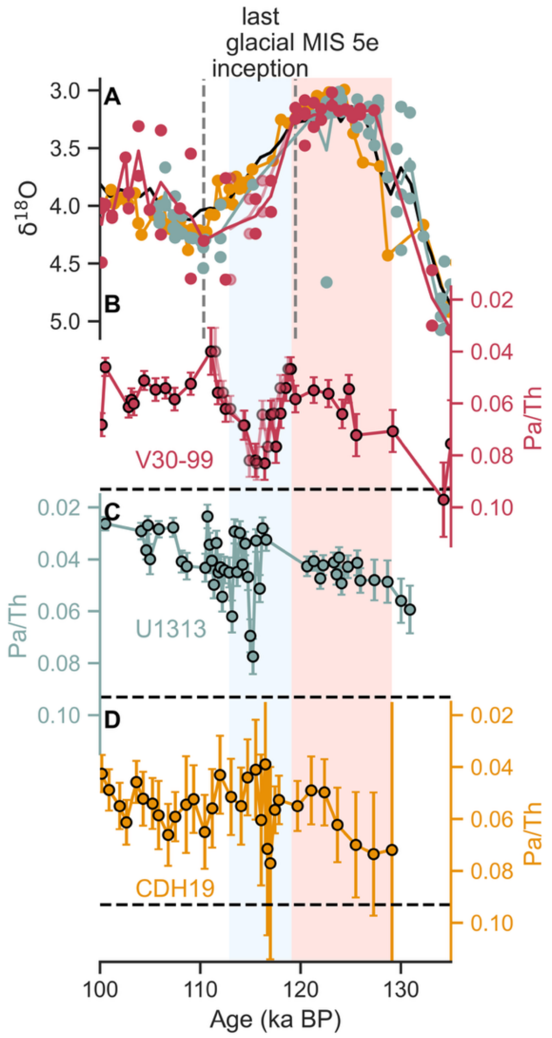

Supplementary Fig. 2. Benthic foraminifera $\delta^{18}O$ and Pa/Th results, showing an alternative V30-99 age model. (**A**) Benthic foraminifera $\delta^{18}O$ results. The colored dots are individual measurements. The colored lines are the three-point moving average. The black line is LR04. The vertical dashed lines marked the tie points used for the construction of the alternative V30-99 age model based on ^{230}Th-excess. (**B**-**D**) Pa/Th results with the 2σ error bars. Notice the y-axes are upside down. The horizontal dashed lines are the Pa/Th production (0.093). The blue shading is the last glacial inception (113-119 ka). The pink shading is the last interglacial (119-129 ka). In (**A**) and (**B**), an alternative V30-99 age model based on ^{230}Th-excess is shown in dark red. The age model as shown in Fig. 2B and 2C is in light red.

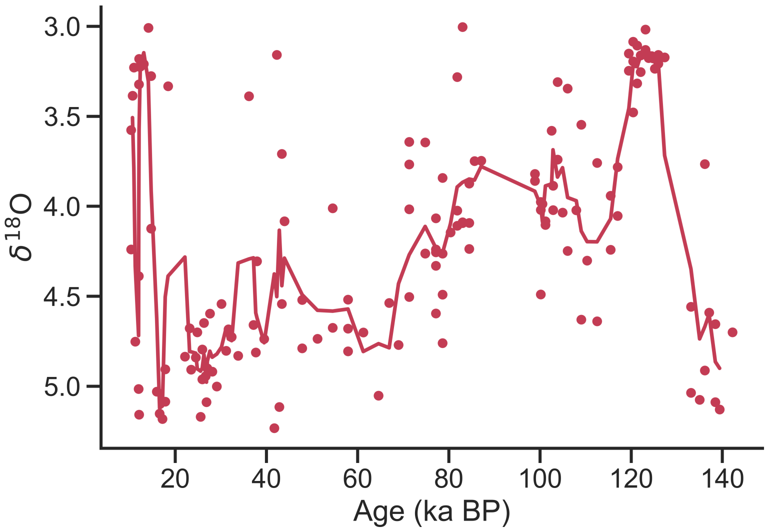

Supplementary Fig. 3. Benthic $\delta^{18}O$ from V30-99 for the last glacial cycle. The line is the three-point moving average of the raw data (dot). The age model is constructed with BIGMACS.

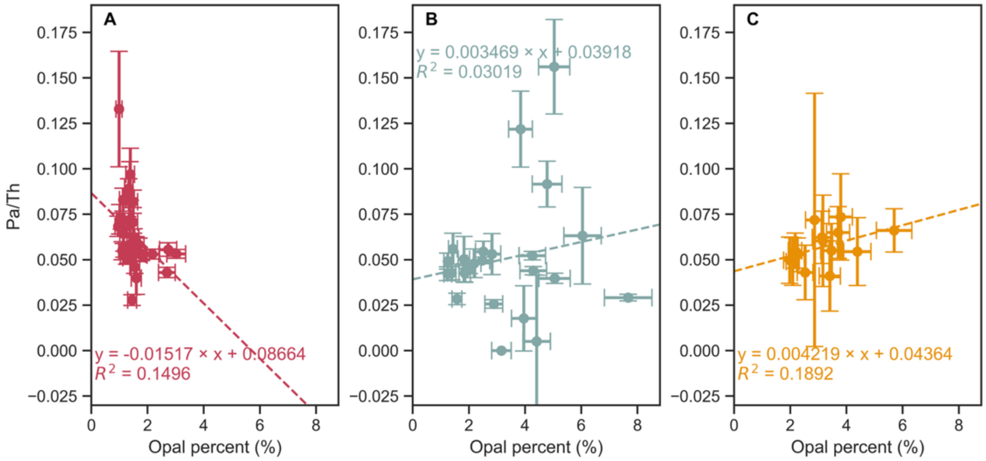

Supplementary Fig. 4. Scatter plots of opal content and Pa/Th in the three cores of our study, V30-99 (**A**), U1313 (**B**), and CDH19 (**C**). The inset equations are the least square linear regression fit and the associated uncertainty.

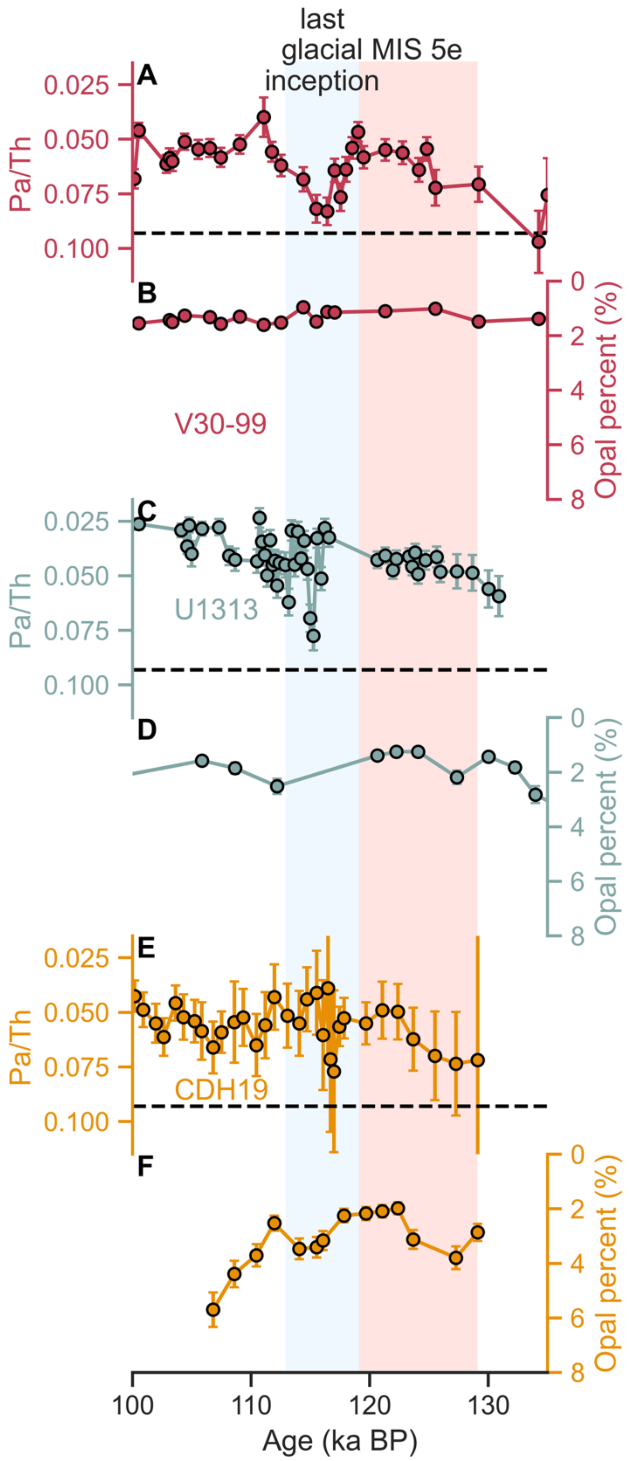

Supplementary Fig. 5. Pa/Th and opal time series comparison. (**A**, **C**, **E**) Pa/Th data during the LGI. (**B**, **D**, **F**) Opal content during the same period.

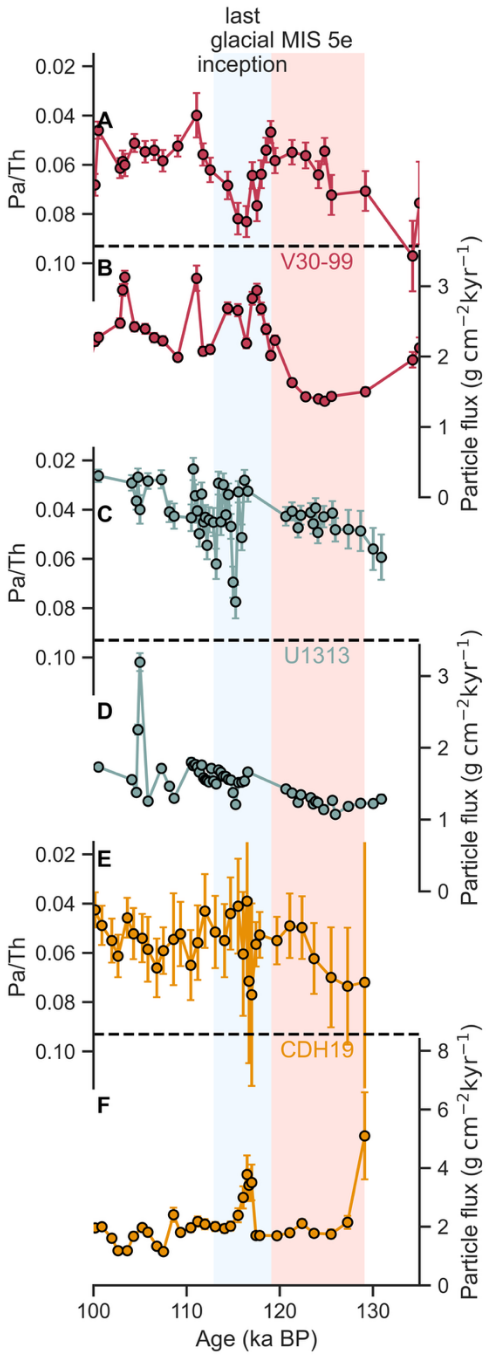

Supplementary Fig. 6. Pa/Th and particle flux results. (**A**, **C**, **E**) Pa/Th data during the LGI. (**B**, **D**, **F**) Particle flux during the same period.

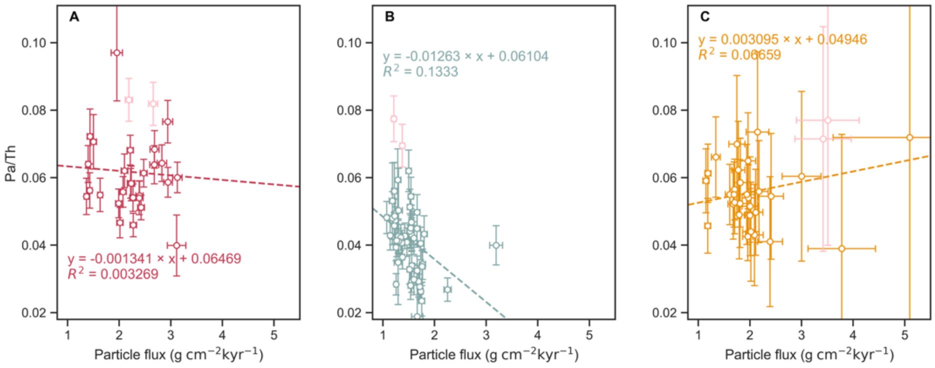

Supplementary Fig. 7. Scatter plots of particle flux and Pa/Th in the three cores of our study, V30-99 (**A**), U1313 (**B**), and CDH19 (**C**). The inset equations are the least square linear regression fit and the associated uncertainty. The pink symbols in each plot are the data points during the LGI interval.

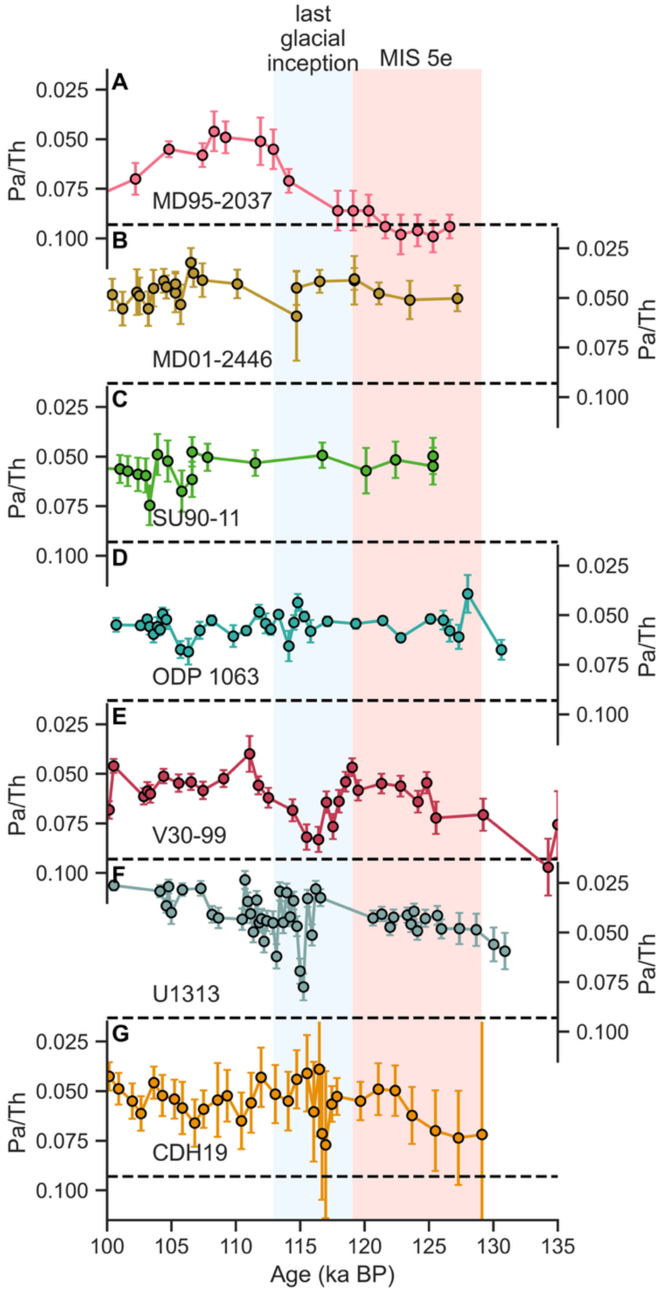

Supplementary Fig. 8. Comparison of Pa/Th records from the literature and this study. (**A**) MD95-2037[1]. (**B**) MD01-2446[2]. (**C**) SU90-11[2]. (**D**) ODP 1063[3]. (**E-G**) records from this study.

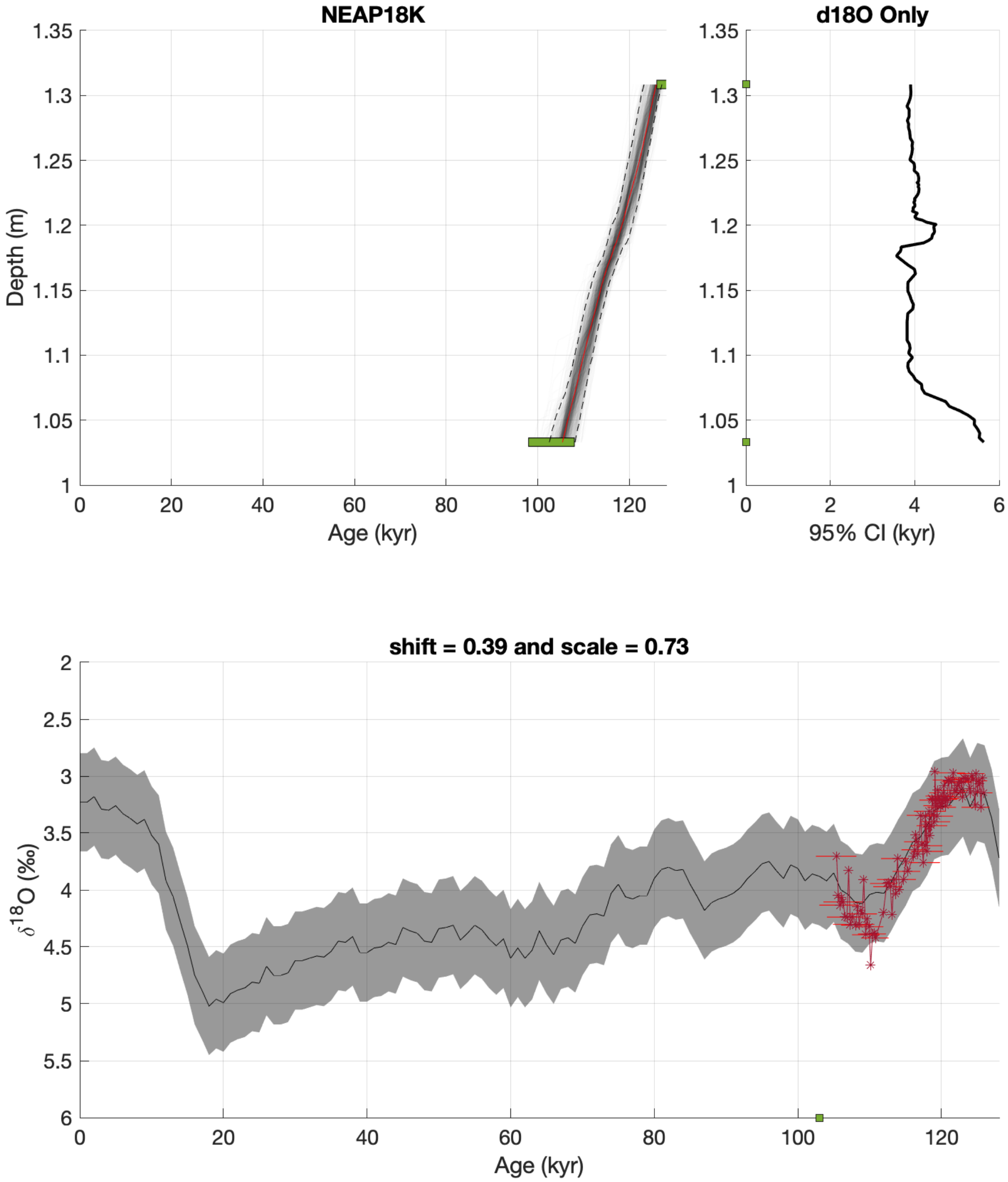

Supplementary Fig. 9. The results of the probabilistic alignment of NEAP18K benthic $\delta^{18}O$ (Hall et al., 1998) to LR04 using BIGMACS. (**A**) Age-depth model shaded according to sample density. The median age model and 95% confidence intervals are plotted as a solid red line and dashed black lines, respectively. Additional ages are plotted as horizontal green rectangles. (**B**) The 95% confidence intervals vs depth with additional ages marked with green squares. (**C**) NEAP18K benthic $\delta^{18}O$ shifted, scaled, and aligned (red stars) with 95% error bars (red lines). The shift and scale parameters are displayed in the panel title. LR04 and its 2σ uncertainty is displayed in the solid black line and gray shade, respectively. Additional ages are denoted with green squares.

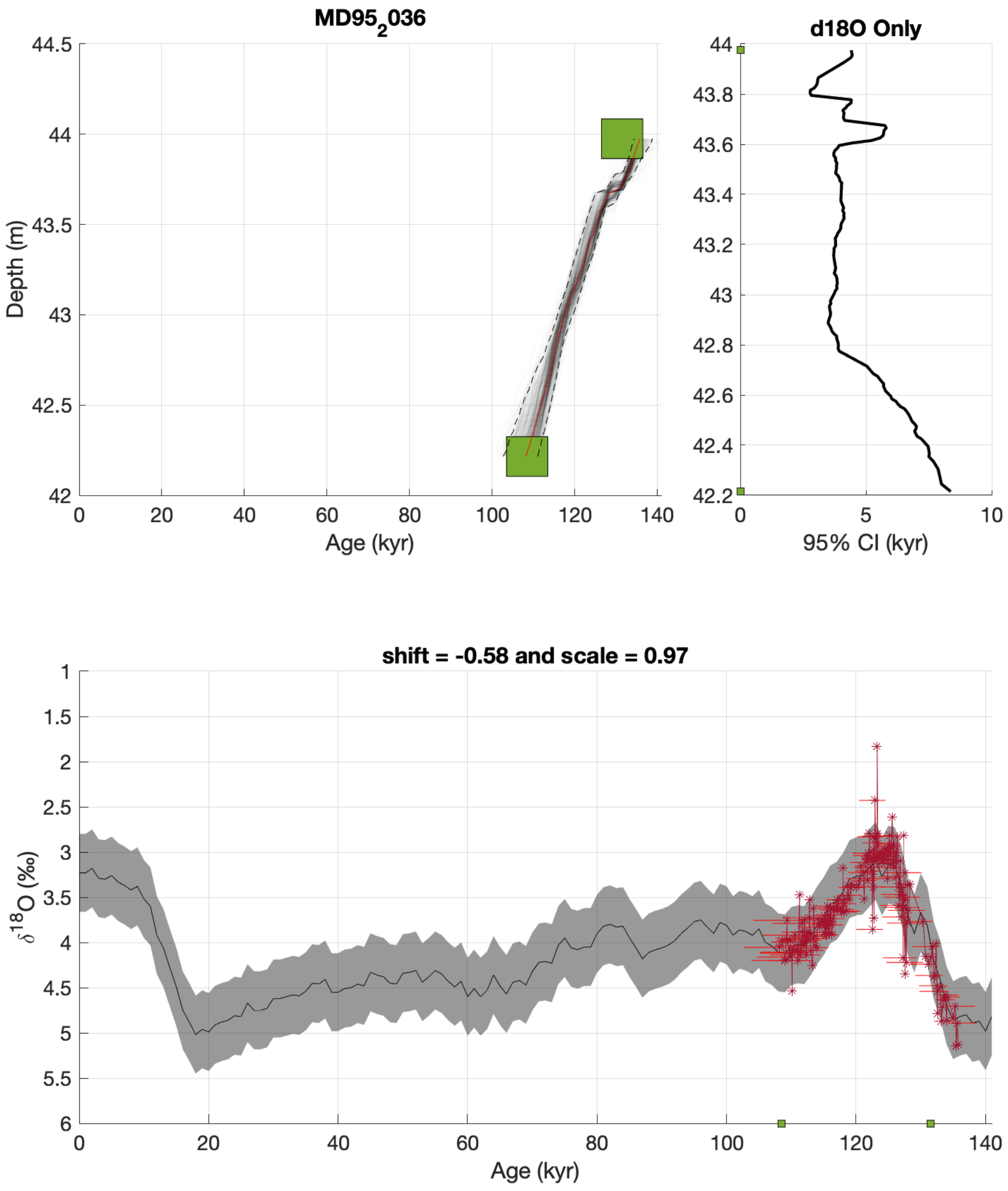

Supplementary Fig. 10. The results of the probabilistic alignment of MD95-2036 benthic δ^{18}O (Adkins et al., 1997) to LR04 using BIGMACS. (**A**) Age-depth model shaded according to sample density. The median age model and 95% confidence intervals are plotted as a solid red line and dashed black lines, respectively. Additional ages are plotted as horizontal green rectangles. (**B**) The 95% confidence intervals vs depth with additional ages marked with green squares. (**C**) MD95-2036 benthic δ^{18}O shifted, scaled, and aligned (red stars) with 95% error bars (red lines). The shift and scale parameters are displayed in the panel title. LR04 and its 2σ uncertainty is displayed in the solid black line and gray shade, respectively. Additional ages are denoted with green squares.

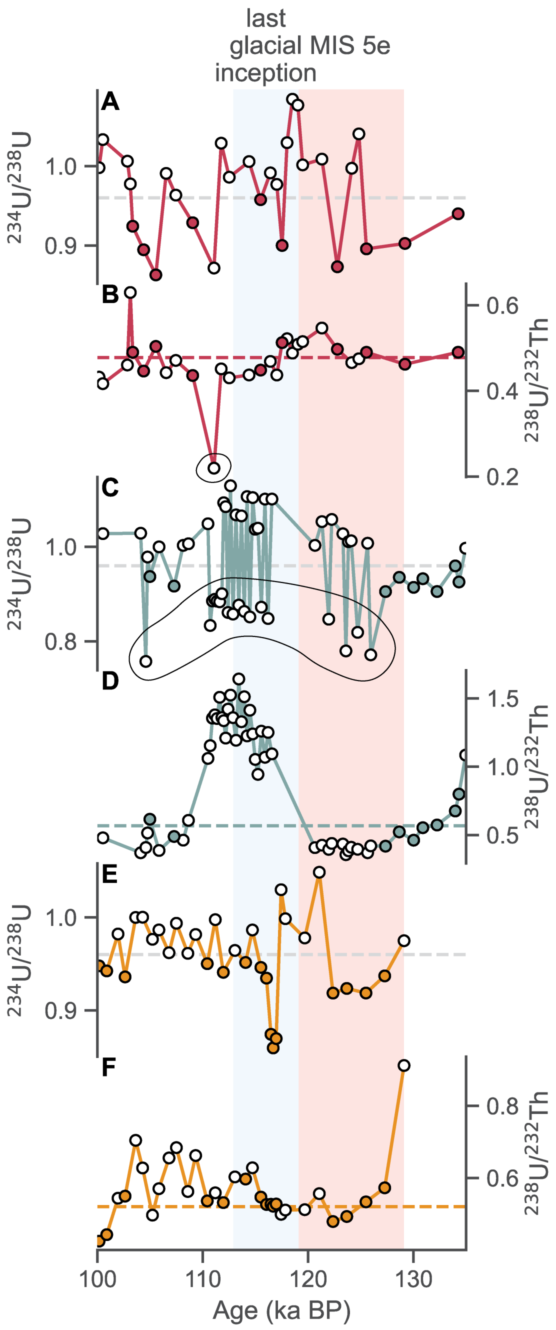

Supplementary Fig. 11. $^{234}U/^{238}U$ and $^{238}U/^{232}Th$ in the three cores of our study, V30-99 (A, B), U1313 (C, D), and CDH19 (E, F). Solid data points are those included in the detrital $^{238}U/^{232}Th$ calculation. Hollow data points are excluded. The horizontal dashed gray lines in (A), (C), and (E) are the $^{234}U/^{238}U$ threshold used to detect authigenic U. The data point in circle in (B) is excluded in the detrital $^{238}U/^{232}Th$ calculation because its $^{238}U/^{232}Th$ value is an outlier. Data points in circles in (C) have their associated $^{238}U/^{232}Th$ values excluded in the detrital $^{238}U/^{232}Th$ calculation because they are from a batch of samples with abnormally low ^{234}U. The horizontal dashed colored lines in (B), (D), and (F) are the resulting detrital $^{238}U/^{232}Th$.

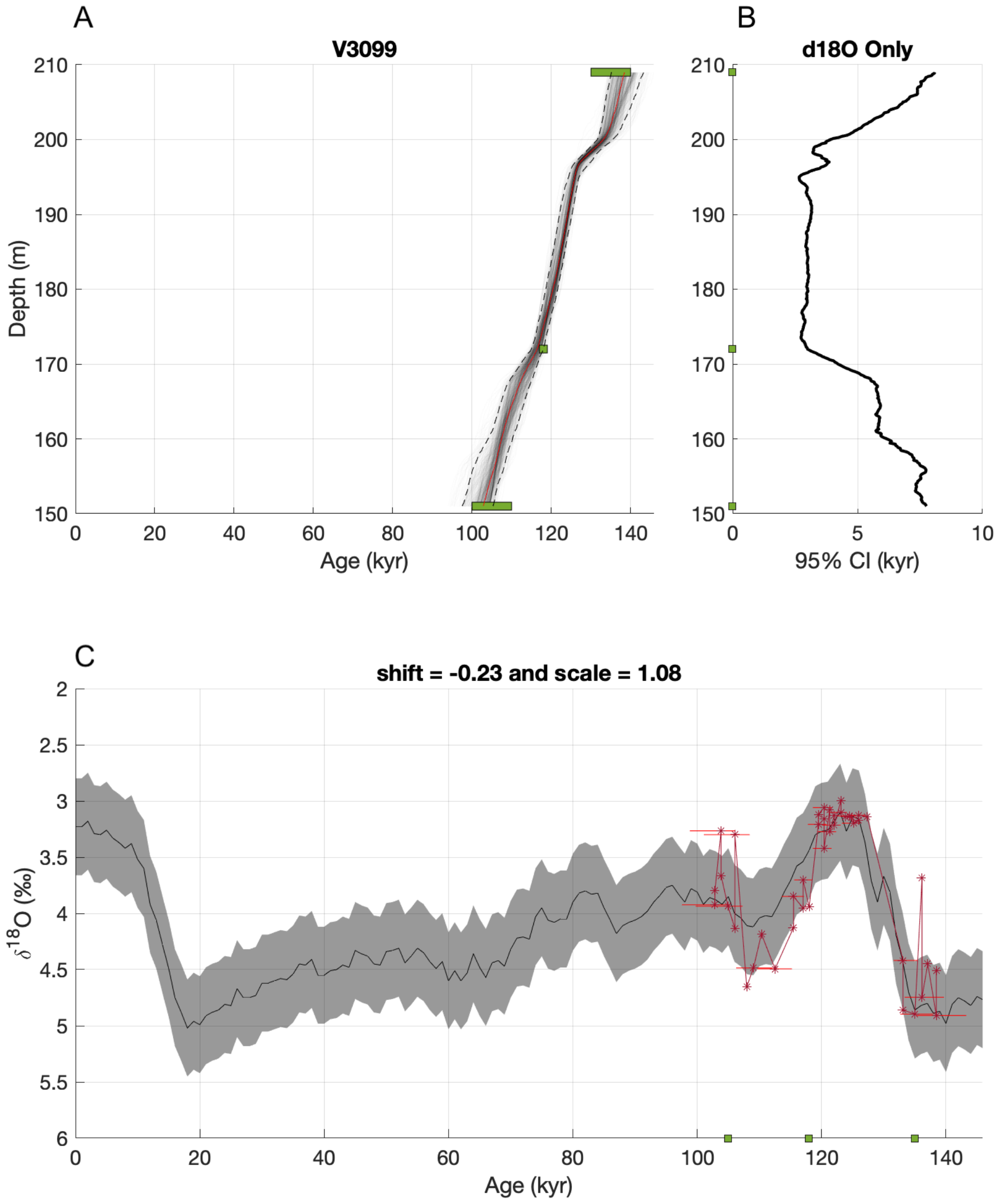

Supplementary Fig. 12. The results of the probabilistic alignment of V30-99 benthic $\delta^{18}O$ to LR04 using BIGMACS. (**A**) Age-depth model shaded according to sample density. The median age model and 95% confidence intervals are plotted as a solid red line and dashed black lines, respectively. Additional ages are plotted as horizontal green rectangles. (**B**) The 95% confidence intervals vs depth with additional ages marked with green squares. (**C**) V30-99 benthic $\delta^{18}O$ shifted, scaled, and aligned (red stars) with 95% error bars (red lines). The shift and scale parameters are displayed in the panel title. LR04 and its 2σ uncertainty is displayed in the solid black line and gray shade, respectively. Additional ages are denoted with green squares.

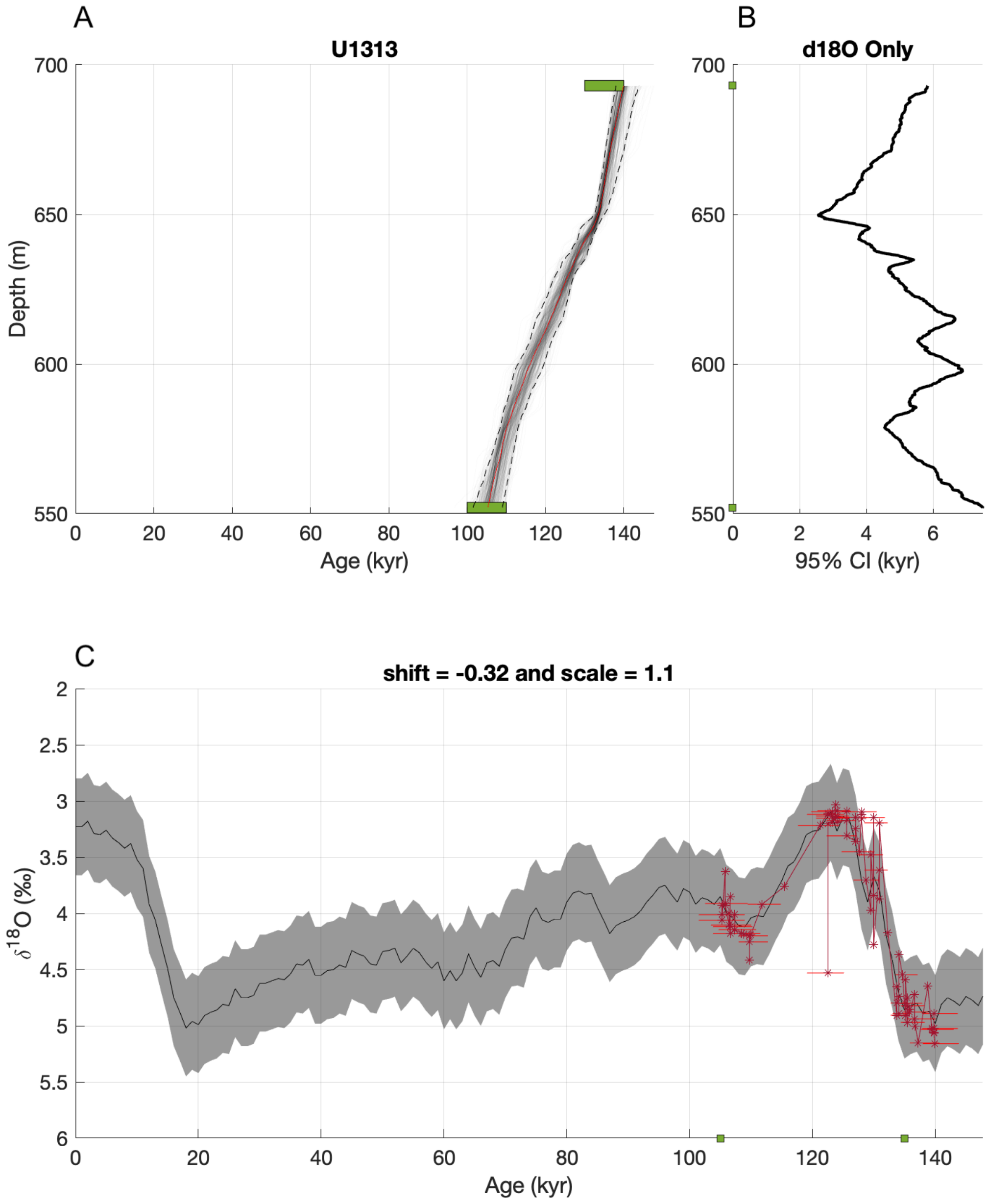

Supplementary Fig. 13. The results of the probabilistic alignment of U1313 benthic $\delta^{18}O$ to LR04 using BIGMACS. (**A**) Age-depth model shaded according to sample density. The median age model and 95% confidence intervals are plotted as a solid red line and dashed black lines, respectively. Additional ages are plotted as horizontal green rectangles. (**B**) The 95% confidence intervals vs depth with additional ages marked with green squares. (**C**) U1313 benthic $\delta^{18}O$ shifted, scaled, and aligned (red stars) with 95% error bars (red lines). The shift and scale parameters are displayed in the panel title. LR04 and its 2σ uncertainty is displayed in the solid black line and gray shade, respectively. Additional ages are denoted with green squares.

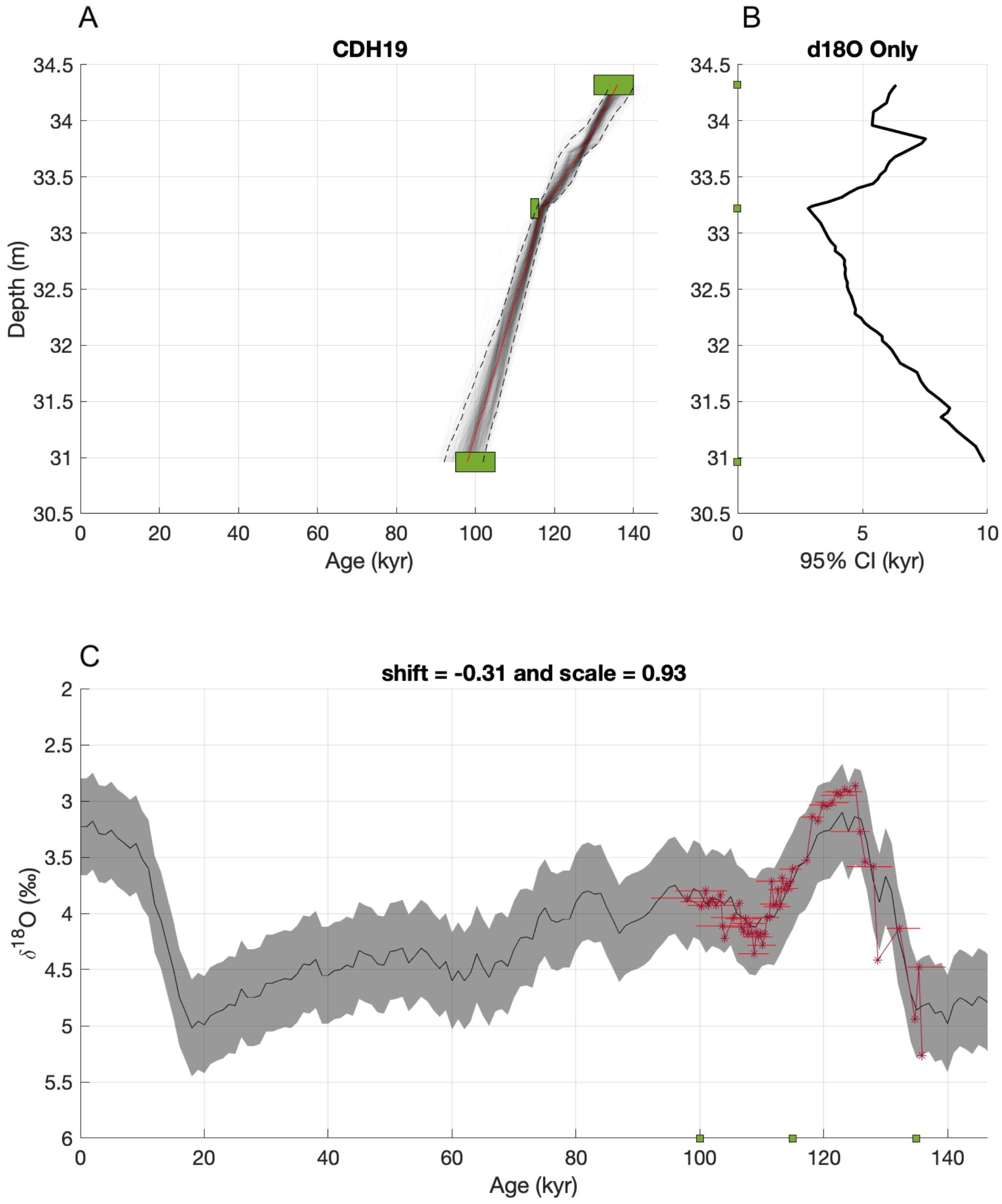

Supplementary Fig. 14. The results of the probabilistic alignment of CDH19 benthic $\delta^{18}O$ to LR04 using BIGMACS. (**A**) Age-depth model shaded according to sample density. The median age model and 95% confidence intervals are plotted as a solid red line and dashed black lines, respectively. Additional ages are plotted as horizontal green rectangles. (**B**) The 95% confidence intervals vs depth with additional ages marked with green squares. (**C**) CDH19 benthic $\delta^{18}O$ shifted, scaled, and aligned (red stars) with 95% error bars (red lines). The shift and scale parameters are displayed in the panel title. LR04 and its 2σ uncertainty is displayed in the solid black line and gray shade, respectively. Additional ages are denoted with green squares.

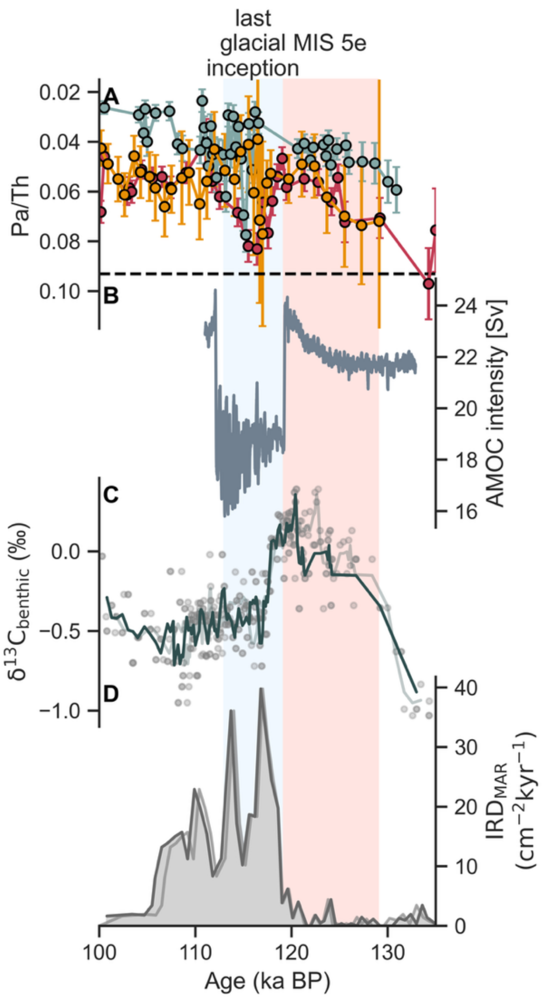

Supplementary Fig. 15. Comparison of the literature records in their respective age model and the LR04-aligned age model. (**A**) Pa/Th records from this study following the same color scheme as Fig. 2. (**B-D**) Same as Fig. 2H-J but in darker colors, the records are aligned to the LR04 target using the same methodology as this study using BIGMACS. In lighter colors, the age models from the respective studies as in Fig. 2 are used.

References

1. Guihou, A. *et al.* Enhanced Atlantic Meridional Overturning Circulation supports the Last Glacial Inception. *Quaternary Science Reviews* **30**, 1576–1582 (2011).

2. Guihou, A. *et al.* Late slowdown of the Atlantic Meridional Overturning Circulation during the Last Glacial Inception: New constraints from sedimentary (231Pa/230Th). *Earth and Planetary Science Letters* **289**, 520–529 (2010).

3. Böhm, E. *et al.* Strong and deep Atlantic meridional overturning circulation during the last glacial cycle. *Nature* **517**, 73–76 (2015).
